# Frequency of Intron 22 Inversion in Severe Hemophilia A Patients

**DOI:** 10.7759/cureus.28247

**Published:** 2022-08-21

**Authors:** Javeria Ashfaq, Rehana Ahmed, Faryal Tariq, Qurat ul Abedin, Madiha Abid, Munira Borhany

**Affiliations:** 1 Clinical Hematology, National Institute of Blood Diseases and Bone Marrow Transplantation, Karachi, PAK; 2 Hematology, National Institute of Blood Diseases and Bone Marrow Transplantation, Karachi, PAK

**Keywords:** factor viii, inhibitor, severity, intron 22 inversion, haemophilia a

## Abstract

Objective: The aim of our study was to find the frequency of Intron 22 inversion (Inv22) in severe hemophilia A (HA) patients and to evaluate the association between Inv22 and FVIII inhibitor formation.

Method: Data analysis was carried out on IBM SPSS Statistics Version 23.00 (IBM Corp, Armonk, NY). Descriptive statistics were applied to measure the frequencies, percentages, and mean ± SD of the clinical and general history of HA patients, including age, family history, inhibitor status, intron22 inversion, and FVIII levels. Chi-square was applied to evaluate the association between Inv22 and F8 inhibitor formation.

Results: A total of 62 HA patients were enrolled in the study with mean±SD age of (14.39±13.2) years. A family history of HA was observed in 36 (58.1%) patients. Out of 62 patients, 28(45.2%) were reported as Inv22 positive while inhibitor status was observed as positive in three (4.83%) patients. However, an insignificant association was observed between the inhibitor and Inv22 positive patients with a p-value=0.443.

Conclusion: In our study, Inv22 was found to be the major cause of severe HA in our patients, i.e., 45.1%. However, no significant relation was computed between Inv22 and inhibitor formation.

## Introduction

The most prevalent blood condition induced by a factor VIII shortage or malfunction is called hemophilia A (HA). It is assumed that HA is a recessive trait linked to the X chromosome [1]. HA was defined as mild with>5% FVIII activity, moderate with 1%-5% FVIII activity, or severe with <1% FVIII activity. Intron 22 inversion (Inv22) is the most prevalent genetic abnormality that causes severe HA (45% of patients) [2]. Others include missense mutation, nonsense mutation, frame splicing, and insertion mutation [3]. Inv22 causes 50% of severe HA cases. Another essential genetic anomaly found in 2%-5% of patients with severe HA is also due to inversion of intron 1 (Inv1). The FVIII gene has a 1041-bp region (Int1h-1) along with its 1 extragenic copy in Intron 1 (Int1h-2; 140 kb telomerically). The recombination of chromosomes between extragenic copy, Int1h-2, and Int1h-1 results in factor FVIII Inv1 [4]. Due to this inversion change, messenger RNA (mRNA) for FVIII does not form, resulting in the absence of FVIII, causing severe HA [5-9]. Both Inv22 variants have been postulated to have a higher likelihood of inhibitor formation, making treatment more difficult [5].

Several methods are used to find changes in HA. Methods like mutation screening and linkage analysis are used to see a specific gene section. Along with these, direct sequencing of the entire gene is also used [10]. Due to its high cost, direct sequencing is uncommon. Direct mutation detection is preferable to indirect linkage analysis; nevertheless, due to limited resources in developing countries like Pakistan, there are some issues with its application. Our goal was to determine the frequency of Inv22 in severe HA patients and the relationship between inhibitor formation and Inv22s.

## Materials and methods

A single-center, cross-sectional study was conducted at the National Institute of Blood Diseases and Bone Marrow Transplantation, Karachi, Pakistan. From November 2019 to December 2021, patients diagnosed with severe HA were screened for Inv22. For data collection, a convenient sampling technique was considered. The sample size was calculated using the WHO calculator.

The Institutional Review Board/Ethics Committee, Hematology, NIBD (approval number NIBD/RD-198/09-2019) approved the study. Informed consent was obtained from all patients. Only severe HA cases were included in the study. While mild, moderate HA cases and patients with other bleeding disorders were omitted.

Genomic DNA (2 µg) was digested with 20 units of BclI in a 50 µL reaction for 4 h. To isolate digested DNA, we utilized phenol-chloroform and ethanol precipitation. T4 DNA Ligase (Invitrogen, Buenos Aires, Argentina) was used to circularise DNA fragments in 400 µL overnight at 15 °C. Next, ligated DNA was treated with equal volumes of a mixture of chloroform and phenol. After removing the aqueous phase, the ethanol precipitated DNA was collected in 30 µL of distilled water. Polymerase Chain Reaction (PCR) was performed in reactions. 0.5 U of Taq DNA polymerase (Promega, Buenos Aires, Argentina) in the presence of 0.6µM of each primer for a total volume of 25 µL. The primer sequences are listed in Table 1. The thermal cycling process includes initial denaturation for 2 minutes at 94°C. Then approximately 30 cycles of denaturation at 94.0 °C (30 sec) with annealing of the primers (60 sec) at 56.0 °C and extension of 90 sec at 72 °C; finally followed by 5 minutes at 72 °C were part of the process. After PCR, the products were run on 2% agarose gel for 30 mins, and under UV light, an image was prepared [11].

**Table 1:** Primer sequence for intron 22 inversion

Primer	Sequence 5′ to 3′	NC_000023.9	BclI site
ID	ACATACGGTTTAGTCACAAGT	153758587-608	27
ED	TCCAGTCACTTAGGCTCAG	154257328-47 154349067-86	99 99
IU	CCTTTCAACTCCATCTCCAT	153779730-50	460
2U	ACGTGTCTTTTGGAGAAGTC	154270775-95	358
3U	CTCACATTGTGTTCTTGTAGTC	154333426-48	306

Data analysis was carried out on IBM SPSS Statistics Version 23.00 (IBM Corp, Armonk, NY). Descriptive statistics were applied to measure the frequencies, percentages, and mean ± SD of the clinical and general history of HA patients, including age, family history, inhibitor status, Inv22, and FVIII levels. Chi-square was applied to evaluate the association between Inv22 and FVIII inhibitor formation.

## Results

A total of 62 HA patients were observed in the study with mean±SD age of (14.39±13.2) years; moreover, age was further categorized into subgroups, i.e., group-I (1-10), group-II (11-20), group-III, (21-30), and group-IV (≥31) years. Most patients belong to group-I (1-10) years of age with 53.2% as compared to the other groups. Positive family history of hemophilia was observed in 36 (58.1%) patients. Out of 62 patients, 28 (45.2%) were reported as Inv22 positive while inhibitor status was observed in three (4.83%) patients whereas 59 (95.2%) patients were without inhibitor as presented in Table 2.

**Table 2:** Demographic Characteristics of Hemophilia A patients

Variables	Frequency (%)
Age Groups (Yrs)	
1-10	33 (53.2)
11-20	12 (19.4)
21-30	12 (19.4)
≥31	5 (8.1)
Family History	
Present	36 (58.1)
Absent	26 (41.9)
FVIII Inhibitor	
Present	3 (4.8)
Absent	59(95.2)
Intron 22 Inversion	
Present	28 (45.2)
Absent	34 (54.8)

Table 3 shows the association of FVIII inhibitor with Inv22. 7.1% of patients with Inv22 had FVIII inhibitors, while 92.9% of Inv22 positive HA patients did not have FVIII inhibitors. Insignificant association, i.e. (p-value=0.443), was observed between FVIII inhibitor and Inv22s as displayed in Table 3.

**Table 3:** Association of FVIII inhibitor with Intron 22 inversions Note: p-value >0.05 indicates insignificant association

Intron 22 Inversion	FVIII Inhibitor		
	Present	Absent	p-value
Present	2 (7.1)	26 (92.9)	0.443
Absent	1 (2.9)	33 (97.1)	

**Figure 1:**
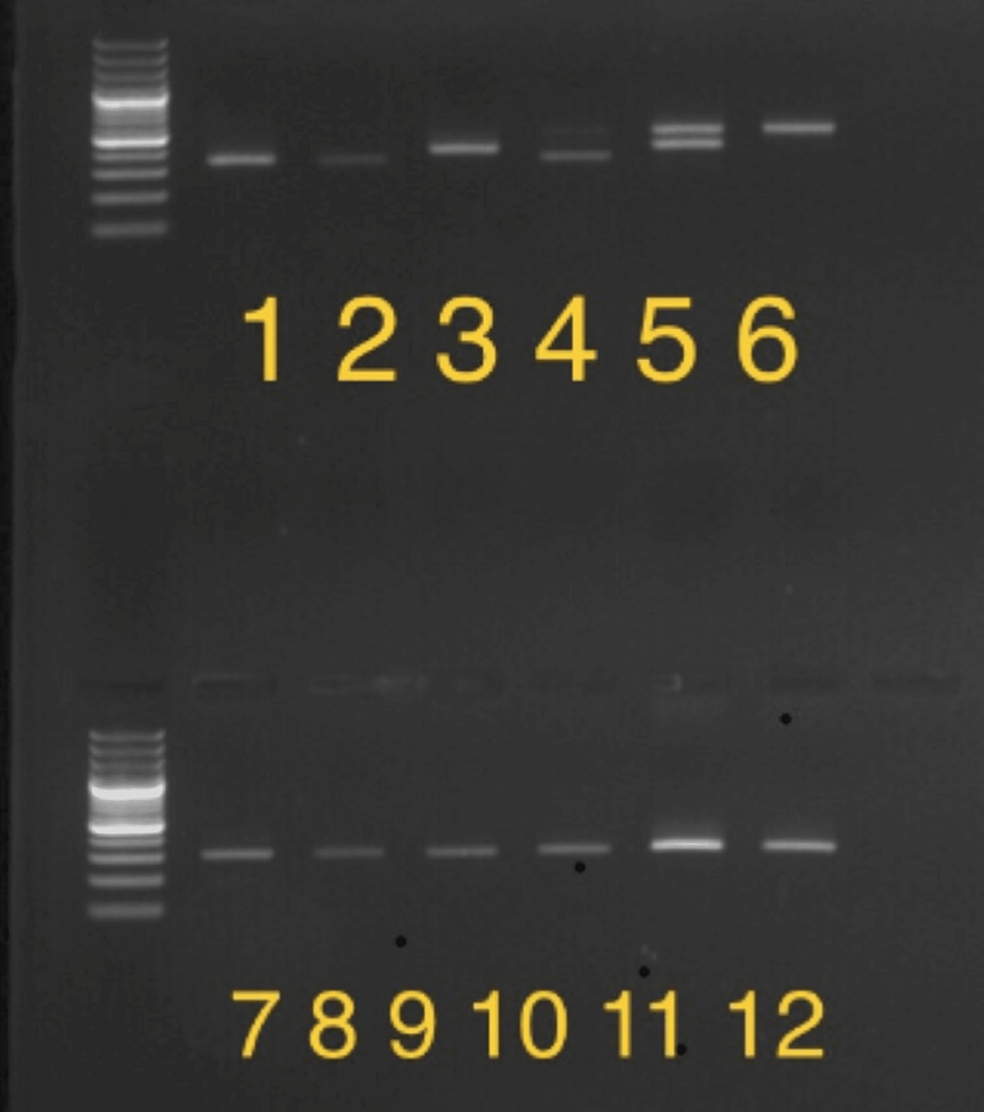
Inversion of FVIII-intron 22 identified in FVIII gene of patients 1 - Int.22 inversion type 1 (Inv22-1)    333bp 2 - Positive control Int.22 inversion type 1 Homozygous (Inv22-1)    333bp 3 - Positive control Int.22 inversion type 2 Homozygous (Inv22-1)    385bp 4 - Positive control Int.22 inversion type 1 heterozygous (Inv22-1)    487; 333bp 5 - Positive control Int.22 inversion type 2 heterozygous (Inv22-1)    487; 385bp 6 - Wild type (Negative control) 487bp 7-12 - Inversion 1 (Negative) 304bp

Figure 1 shows inverse shifting PCR (IS-PCR) analyses for FVIII intron 22 related inversions using 1% agarose gels. Genetic counseling and genetic studies are advised. Inversion of FVIII intron 22 is responsible for 45%-50% of severe HA. This test also represents the method of choice at the first line in severe HA patients.

## Discussion

Hemophilia is an X-linked inherited bleeding illness having a very high treatment cost, and developing-country governments do not place a high priority on technology-intensive therapy. A recent Pakistani study on Congenital Hemorrhagic Disorders Group by Sajid et al. [12] and other local studies have shown that HA is the most common bleeding disorder in the Pakistani population. Factor replacement therapy for the treatment of patients with hemophilia and other bleeding disorders is mainly based on fresh frozen plasma and its components. Our findings revealed that 58.1% of patients had a positive family history. These findings highlight the value of early screening of newborns whose families are at high risk of the disease. 40%-50% of individuals worldwide have the disease due to Inv22 mutation. According to Abu Arra et al., this is the first line of testing in present individuals [2]. The positive proportion of Inv22 in our study was 45.2%. This positive frequency is similar to previous studies from Egypt (46.1%), Iran (47%), and Iraq (36.3%) but lower than Jordan (52%) and Saudi Arabia (50%). It is higher than Albania (10.5%), Tunis (22.7%), Lebanon (29%), and the United Kingdom (17.6%) [13-18]. We detected inhibitors in 7.1% of Inv22 positive patients during our research.

According to statistics from the previously published study [19,20], the inhibitors' prevalence in people with inversions of intron 22 was different. Goodeve et al. and Astremark et al. [21,22] identified the changes in the FVIII gene, especially Inv22, as a significant component that increases the development of inhibitors in patients suffering from a severe form of the disease. According to the current study, inversion of intron 22 is our population's most common change in severe HA. Inv22 is the essential genetic component for inhibitors' development. However, our study did not show the association between Inv22 and inhibitors' development. 

In India, Ghosh et al. [23] found an inhibitor frequency of 8.2% in individuals with severe HA; however, 24% of Inv22 positive patients developed an inhibitor. Ghosh et al. proposed that treating patients regularly causes the inhibitors to withdraw from blood circulation during treatment sessions, which lowers the prevalence frequency, especially temporary inhibitors. Borhany et al. [24] did a study in Pakistan and found that 15% of hemophilia A patients had inhibitors. There is also a natural history or development of a temporary low-level inhibitor for hemophilia treated with factor VIII.

There are limited data on this topic in the Pakistani population, and the small sample size is a limitation of this study. The current study is a single-center study; therefore, multi-center longitudinal studies should be conducted in the future.

## Conclusions

Inv22s are a major cause of severe HA in Pakistani individuals. Inv22s were found in 45.2% of severe hemophilia A patients in our study. Despite being linked to severe HA phenotypic instances, the Inv22 mutation was not a significant risk factor for inhibitor production. Additional research involving more patients is advised, along with testing for additional mutations.
